# The therapeutic potential of exercise in post-traumatic stress disorder and its underlying mechanisms: A living systematic review of human and non-human studies

**DOI:** 10.12688/wellcomeopenres.23033.1

**Published:** 2024-12-07

**Authors:** Simonne Wright, Virginia Chiocchia, Olufisayo Elugbadebo, Ouma Simple, Toshi A. Furukawa, Claire Friedrich, Charlotte Austin, Hossein Dehdarirad, David Gilbert, Jaycee Kennett, Edoardo G. Ostinelli, Jennifer Potts, Fiona Ramage, Emily Sena, Spyridon Siafis, Claire Stansfield, James Thomas, Francesca Tinsdeall, Thomy Tonia, Malcolm Macleod, Andrea Cipriani, Georgia Salanti, Soraya Seedat

**Affiliations:** 1 South African Medical Council Unit on the Genomics of Brain Disorders, Department of Psychiatry, Stellenbosch University Faculty of Medicine and Health Sciences, Cape Town, Western Cape, 7550, South Africa; 2 University of Bern Institute of Social and Preventive Medicine, Bern, Switzerland; 3 Department of Psychiatry, University of Ibadan College of Medicine, Ibadan, Oyo, Nigeria; 4 Makerere University School of Medicine, Kampala, Central Region, Uganda; 5 Kyoto University Office of Institutional Advancement and Communications, Kyoto, Japan; 6 Department of Psychiatry, University of Oxford, Oxford, England, UK; 7 Oxford Precision Psychiatry Lab, NIHR Oxford Health Biomedical Research Centre, Oxford, England, UK; 8 EPPI Centre, UCL Social Research Institute, University College London, London, England, UK; 9 GALENOS Global Experiential Advisory Board, InHealth Associates, London, UK; 10 NIHR Oxford Health Clinical Research Facility, Oxford Health NHS Foundation Trust, Warneford Hospital, Oxford, England, UK; 11 The University of Edinburgh Centre for Clinical Brain Sciences, Edinburgh, Scotland, UK; 12 Technical University of Munich, TUM School of Medicine and Health, Department of Psychiatry and Psychotherapy, Munich, Germany; 13 German Center for Mental Health (DZPG), partner site, München/Augsburg, Germany

**Keywords:** GALENOS, PTSD, exercise, extinction learning, memory regulation, emotion regulation

## Abstract

**Background:**

Exercise for post-traumatic stress disorder (PTSD) is a potentially effective adjunct to psychotherapy. However, the biopsychosocial mechanisms of exercise are not well understood. This co-produced living systematic review synthesizes evidence from human and non-human studies.

**Methods:**

We Included controlled human and non-human studies involving searches of multiple electronic databases (until 31.10.23). Records were screened, extracted, assessed for risk of bias, and reconciled by two independent reviewers. The primary outcome for human studies was PTSD symptom severity, while outcomes of interest for non-human studies included freezing behaviour, fear memory, fear generalization, startle response, and locomotion. Data were synthesised with random-effects meta-analysis.

**Results:**

Eleven human studies met the eligibility criteria. Overall, exercise was not associated with symptom severity improvement compared to control (standardized mean difference [SMD] -0.08, 95% confidence interval [CI] -0.24 to 0.07; 8 studies, one at low risk of bias). High-intensity exercise reduced PTSD symptoms scores more than moderate-intensity exercise. There was insufficient data to examine the effects of exercise on functional impairment, PTSD symptom clusters, and PTSD remission. Only three studies, all at high risk of bias, examined mechanisms of exercise with inconclusive results. Exercise was associated with improvement in all behavioural outcomes, including locomotor activity (SMD 1.30, 95% CI 0.74 to 1.87, 14 studies), and changes in several neurobiological markers, including increase in brain-derived neurotrophic factor (SMD 1.79, 95% CI 0.56 to 3.01).

**Conclusions:**

While non-human studies provide compelling evidence for the beneficial effects of exercise, human trials do not. Evidence from non-human studies suggest that exercise might increase the levels of brain-derived neurotrophic factor, enhance cognitive appraisal, and improve perceived exertion. Overall, the paucity of data on the effectiveness of exercise in PTSD and mechanisms of action underscore the need for rigorous trials.

**Registration:**

The protocol was registered with PROSPERO (ID:453615; 22.08.2023).

## Plain Language Summary

Post-traumatic stress disorder (PTSD) is a mental health condition triggered by a traumatic event. It is characterised by symptoms such as reliving the trauma, nightmares, avoidance of trauma reminders, and heightened vigilance. Traditional treatments like Cognitive Behavioral Therapy (CBT) and Eye Movement Desensitization and Reprocessing (EMDR) are not effective for everyone, and relapse is common. Additionally, these treatments are costly and not widely accessible. Recently, exercise has been explored as a potential stand-alone or add-on treatment to improve PTSD outcomes and reduce relapse rates.

We reviewed evidence from human and non-human studies for the effectiveness of exercise on PTSD symptoms and functioning. We also reviewed how exercise influences symptoms through biological factors, like brain-derived neurotrophic factor (BDNF), and psychosocial factors, such as cognitive appraisal and emotional state. In our search, we identified 11,870 human studies from multiple databases, of which 11 were deemed eligible. Of the human studies, three investigated responses to threat and BDNF levels, BDNF levels, and subjective feelings during exercise, respectively.

For non-human studies, 1,222 records were found across eight databases, with 14 studies qualifying for inclusion. The non-human studies focused on various factors influencing PTSD treatment outcomes, including BDNF (8 studies), stress physiology (6 studies), and other brain and body changes (9 studies).

Non-human studies find that exercise is effective for PTSD. Findings from human studies are not conclusive, although high-intensity exercise shows promise in reducing PTSD symptom severity. How exercise improves PTSD is still not well understood, although there is some evidence that exercise might work by increasing BDNF levels, enhancing cognitive appraisal, and improving perceived exertion. These findings underscore the need for further high-quality research to establish whether exercise enhances the effects of psychotherapy for PTSD and the mechanisms by which it does so.

## Background

Posttraumatic stress disorder (PTSD) is a persistent trauma-related disorder characterised by reexperiencing symptoms, avoidance symptoms, negative cognitions and mood, and hyperarousal symptoms that can cause pervasive distress and functional impairment ^1^. Although trauma-focused therapies are effective evidence-based interventions for PTSD ^2, 3^, not all patients respond and relapse rates post-treatment are high ^4, 5^.

Exercise, defined as structured physical activity, is a cost-effective and accessible way to enhance physical, social, and mental well-being ^6^. It may serve as a non-pharmacological augmentation to psychotherapy or to medication for PTSD, offering benefits such as stress reduction and mood regulation ^7^. A recent systematic review showed that exercise, including walking, jogging, dancing, strength training, and yoga, had moderate effects on depression, as both standalone or adjunctive treatment ^8^.

Although the mechanisms of exercise are not fully understood, several plausible biological pathways have been identified. Exercise induces changes in neurotransmitters, modulators, and peptides that affect neural pathways involved in extinction learning ^9^, a process crucial for inhibiting fear responses in PTSD. These neurochemical processes include the activation of endocannabinoids ^10^, dopaminergic signalling ^11^, mammalian target of rapamycin (mTOR; 12), and brain-derived neurotrophic factor (BDNF; 13). Increased BDNF levels have been linked to improved neuroplasticity and enhanced fear extinction in individuals with PTSD, highlighting the potential of exercise to alleviate symptoms ^14, 15^.

Beyond biological mechanisms, psychosocial factors also contribute to the therapeutic effects of exercise in PTSD. Exercise can enhance social interaction, self-efficacy, and distraction from intrusive symptoms, which are all beneficial for PTSD treatment ^16^. Social support, fostered through group exercise, has been shown to reduce PTSD symptom severity ^17, 18^.

The single prolonged stress (SPS) model, frequently used in non-human studies and has also been used to elucidate the effects of exercise on PTSD and its underlying mechanisms ^19^. The SPS model mirrors human PTSD as it imitates the traumatic stressors experienced ^20^. Although both SPS and PTSD are characterized by traumatic exposures, the distinction lies in the intensity and persistence of symptoms ^21^. In PTSD, traumatic experiences produce a long-lasting effect, whereas the impact in SPS is acute ^22, 23^. Investigating these mechanisms through exercise could open pathways for more precise PTSD treatments, potentially leading to improved clinical outcomes.

### Review objectives

The objective of this study is to review the evidence on the effects of exercise in PTSD in humans and non-human models and elucidate how exercise mechanistically reduces symptom severity and/or functional impairment in PTSD.

## Methods

This is the first iteration of a living systematic review of human and non-human studies under the umbrella of the Global Alliance for Living Evidence on aNxiety, depressiOn, and pSychosis ( GALENOS) project ^24^. The protocol was developed following the GALENOS protocol template for ^25^, published in Wellcome Open Research ^26^, and registered with PROSPERO (ID:453615; 22.08.2023) and Open Science Framework ( Extended data). Reporting is done according to PRISMA ( 27; Extended data). Below, we summarised the key methodological aspects; for a detailed methods description see 26.

### Eligibility criteria

We included randomized and non-randomized controlled trials with participants with either above-threshold symptoms on any standardized self-report measure or a clinical diagnosis of PTSD. We included studies comparing (i) exercise versus inactive comparison group/sham intervention, or (ii) exercise group + psychotherapy versus psychotherapy alone (the aspects of psychotherapy had to be identical). We excluded exercise interventions that incorporated mindfulness (e.g., Pilates, martial arts, yoga, tai chi, and horseback riding) or psychotherapy-based techniques into the exercise protocol.

For the review of non-human studies, we included controlled experimental studies that modelled PTSD using Single Prolonged Stress (SPS).

### Outcomes

For human studies, the primary outcome was PTSD symptom severity. Secondary outcomes were functional impairment (e.g., disability, quality of life), avoidance symptom severity, reexperiencing symptom severity, hyperarousal symptom severity, negative cognitions and mood severity, PTSD remission, threat expectancy ratings, and study dropout. For non-human studies, the outcomes of greatest interest were freezing behaviour, fear memory, fear generalization, increased startle response, locomotion activity, and sleep electroencephalography (EEG).

### Study identification

Search strategies for human and non-human studies are reported in the Extended data. For human studies, CS conducted the searches limited to English publications in Biosis (WOS), CENTRAL (Cochrane Library), CINAHL (nursing and allied health) (EBSCO), Embase (OVID), MEDLINE (OVID), PsycInfo (OVID), PTSDpubs (PROQUEST), Scopus, Web of Science Core Collection (until 30.08.2023). HD conducted searches in ClinicalTrials.gov, SanMedicine and WHO-ICTRP (until 31.10.2023).

For non-human studies, FR and MM conducted searches in PubMed, Web of Science, Scopus, and PsycInfo (until 24.08.23). We also searched for unpublished non-human studies in registries ( animalstudyregistry.org, preclinicaltrials.eu) and preprint databases (medRxiv, bioRxiv).

### Study selection and data extraction

Each record was screened by two independent reviewers, with differences reconciled by a third (SW, OE, OS, CF, and SS for human studies; FT, FR, CA, CF, JK, OM, and MRM for non-human studies). For human studies we used the EPPI reviewer ^28^ and for non-human studies the SyRF software (SyRF, RRId SCR_018907) ^29^.

For continuous outcomes, we extracted the mean, standard deviation (SD), and sample size for each assigned group in a study. For dichotomous outcomes, we extracted the numbers from cross-classified tables.

Extraction of data was carried out using techniques that accounted for missing data, prioritized (i) mixed-models of repeated measurement and multiple imputations, followed by (ii) last-observation carried forward, and finally (iii) observed cases. Data were collected at specific time points including at pre-intervention (baseline) and at the primary endpoint, defined as the first post-intervention time point or study endpoint if exercise was given for the entire duration of the trial. We also collected data for all subsequent follow-up assessment time points. We extracted data on potential effect modifiers: exercise intensity (moderate-intensity or high-intensity), specific exercise type (aerobic, anaerobic, or mixed), exercise augmented by treatment-as-usual or psychotherapy, intervention length, and study risks of bias. For non-human studies, additional potential moderators are sex, voluntary vs forced exercise, and study reporting quality.

We also extracted data on potential mediators of the effect of exercise. For human studies these included BDNF, anandamide (AEA), 2-arachidonoylglycerol (2-AG), and homovanillic acid (HVA), cognitive appraisal, perceived exertion, affect, arousal, and distress. For non-human studies, reported mediators could also be brain neurotransmitter levels including dopamine and 5-HT, cell death markers including Bax, Bcl-2 and caspase3, serum corticosterone levels, and dexamethasone suppression tests.

We assessed the risk of bias on primary outcomes using the Cochrane Risk of Bias 2 (RoB2; 30 for randomised human studies, a tool made out of concepts described in ^31^ for mediation studies, and SYRCLE tool ^32^ for non-human studies. We evaluated the completeness of reporting of non-human studies using ARRIVE2.0 ^33^ and for studies on mediation analyses using A Guideline for Reporting Mediation Analyses (AGReMA; 34).

### Data analysis

The data analysis was conducted in R version 4.3.0 for human studies; version 4.3.1 for non-human studies.

For continuous outcomes, we calculated standardized mean difference (SMD); for dichotomous outcomes, we calculated risk ratios (RR).

***Synthesis of data for the effect of exercise***

We conducted a quantitative synthesis via a random effects meta-analysis. To synthesize non-human studies, we conducted multilevel meta-analysis by strain, study, and experiment ^35^, where enough data were available for each level; otherwise, we conducted conventional random effects meta-analysis. Heterogeneity was quantified using the between-study variance τ ^2 ^and presented with the 95% prediction intervals (95% PrI) of the treatment effects. For synthesis of non-human studies, we additionally present the variance attributed to strain, study, and experiment.

When there was insufficient data to conduct a meta-analysis, we present the evidence following the SWiM principles of reporting ^36^. When there were enough studies, we explored the effect of possible effect modifiers on the primary outcome using subgroup analysis or meta-regression. In a post-hoc analysis to gain insight on the animal models, we also analysed the effect of SPS against control (i.e. groups not exposed to SPS).

We performed a sensitivity analysis to assess the impact of restricting the meta-analysis to studies with a low risk of bias for the primary outcome in human studies. For non-human studies we conducted sensitivity analyses (i) using different imputed values for correlations between observations from the same experimental cohort, and (ii) using a normalized mean difference (NMD) rather than SMD.

We examined the presence of small-study effects using a contour-enhanced funnel plot for human studies and a multivariate version of Egger’s regression test in non-human studies.

***Synthesis of data from mediation analyses***

We have planned to synthesise the data from mediation analyses using methods described in our protocol; however, not enough data were available, so we present the findings without a quantitative synthesis (see Extended data for Mediation analyses methods for future iterations).

For non-human studies, changes in neurobiological outcomes (such as BDNF) might be intermediate outcomes, driving any observed change in behaviour (which might be considered ‘apical’ endpoints). We examined the relationship between effects sizes reported for different outcome measures reported from the same experimental cohorts ( Extended data).

### Summary of the evidence

We summarize the evidence using Summary of Evidence (SoE) tables separately for human and non-human studies. The tables include considerations about the (i) bias due to study limitations; (ii) bias due to reporting; (iii) bias due to indirectness; and (iv) bias due to other reasons. For details see the 26.

### Patient and public involvement

The Global Experiential Advisory Board (GLEAB) were involved in the selection of the research question for LSR2 and the chair of GLEAB was involved in co-producing the protocol and the manuscript.

## Results

Detailed report of the human ( Extended data) and non-human studies ( Extended data) analyses are provided. We also provide the summary of evidence tables for human ( Extended data) and non-human studies ( Extended data).

### Evidence from human studies

**Figure 1.**
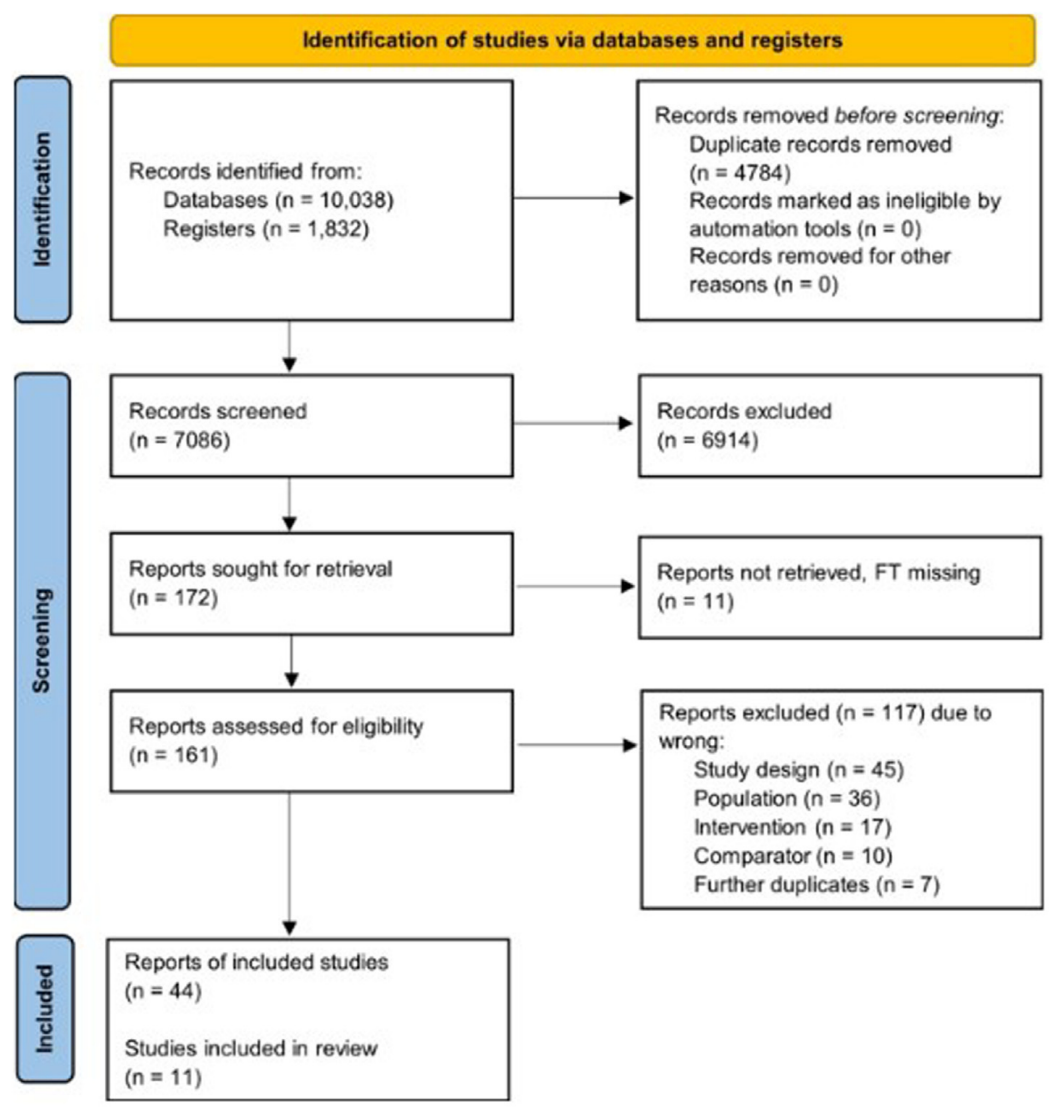
Flow of study selection and descriptives for the human studies.

The PRISMA flow diagram is presented in Figure 1. A total of 11 studies with data from 771 participants were eligible for inclusion. Ten studies were parallel RCTs, and one was a crossover RCT ^37^. The specific intervention and comparison groups for the 11 eligible studies (13 comparisons) are presented in Table 1. Three studies ^37– 39^ provided insufficient outcome data; we reported their comparative effects without a meta-analysis. Three studies examined putative mediators on either PTSD severity or threat expectancy rating ^38– 40^. The eight studies with outcome data provided 9 eligible comparisons; one study presented findings from two independent comparisons ^41^. Results of the RoB for PTSD severity outcome are presented in the Extended data. Five of the nine comparisons had an overall high risk of bias.

**Table 1.** Specific interventions and study characteristics for all studies.

Study	N	Intervention	Comparison	PTSD tool	Trial registered	Exercise type	Exercise intensity	Intervention length	Therapy	TAU	Attention control	FU1	FU2	Country
**Characteristics of studies included in the meta-analysis**														
Bryant (2023)	130	exercise + therapy	attention control + therapy	CAPS-IV	yes	aerobic	high	10 weeks	exposure therapy	NA	static stretching	34 weeks	NA	Australia
Voorendonk (2023)	120	exercise + therapy	attention control + therapy	PCL-5	yes	mixed	moderate	12 weeks	PE + EMDR	NA	guided (creative) tasks	26 weeks	NA	Netherland
Nordbrandt (2020)	224	exercise + TAU	TAU	HTQ	yes	mixed	moderate	20 weeks	NA	combination: medical doctor, 1 to 2 sessions with social worker / psychologist	NA	NA	NA	Denmark
Rosenbaum (2014)	81	exercise + TAU	TAU	PCL-4	yes	mixed	high	12 weeks	NA	combination: individual and group psychotherapy, pharmacotherapy	NA	NA	NA	Australia
Young- McCaughan (2022b)	36	exercise + therapy	therapy only	PCL-5	no	aerobic	high	8 weeks	imaginal exposure	NA	NA	12 weeks	32 weeks	USA
Huseth (2022)	21	exercise only	WLC	PCL-5	no	aerobic	moderate	8 weeks	NA	NA	NA	NA	NA	USA
Young- McCaughan (2022a)	36	exercise only	TAU	PCL-5	no	aerobic	high	8 weeks	NA	self-care intervention delivering educational and instructional information	NA	12 weeks	32 weeks	USA
Whitworth (2019a)	30	exercise only	attention control	PDS-5	no	anaerobic	high	3 weeks	NA	NA	videos on various educational topics (excluding exercise and mental health).	NA	NA	USA
Whitworth (2019b)	22	exercise only	attention control	PDS-5	no	anaerobic	high	3 weeks	NA	NA	videos on various educational topics (excluding exercise and mental health).	NA	NA	USA
**Characteristics of studies not included in the meta-analysis**														
Crombie (2021)	38	exercise + extinction learning	attention control + extinction learning		yes	aerobic	moderate	3 days	extinction learning	NA	NA			USA
Greene (2022a)	24	exercise only	attention control		no	anaerobic	high	130 min	NA	NA	remained sedentary in the lab			USA
Greene (2022b)	NA	exercise only	attention control		no	aerobic	moderate	130 min	NA	NA	remained sedentary in the lab			USA
Powers (2015)	9	exercise + therapy	therapy alone		yes	aerobic	moderate	12 weeks	prolonged exposure	NA	NA			USA

*TAU = treatment as usual; WLC = waiting list control; CAPS-IV = Clinician-Administered PTSD Scale - 4th edition; PCL-4 = PTSD Checklist - version 4; PCL-5 = PTSD Checklist - version 5; PDS-5 = Posttraumatic Diagnostic Scale – version 5; HTQ = Harvard Trauma Questionnaire; Aerobic exercise = physical performance behaviour pattern that increases heart rate and respiration while using large muscle groups repetitively and rhythmically; anaerobic exercise = physical performance behaviour pattern that is performed in short intense bursts with limited oxygen intake; mixed exercise = combination of aerobic and anaerobic exercise; USA = United States of America; FU1 = first follow-up assessment; FU2 = second follow-up assessment.*

### PTSD symptom severity

**Figure 2.**
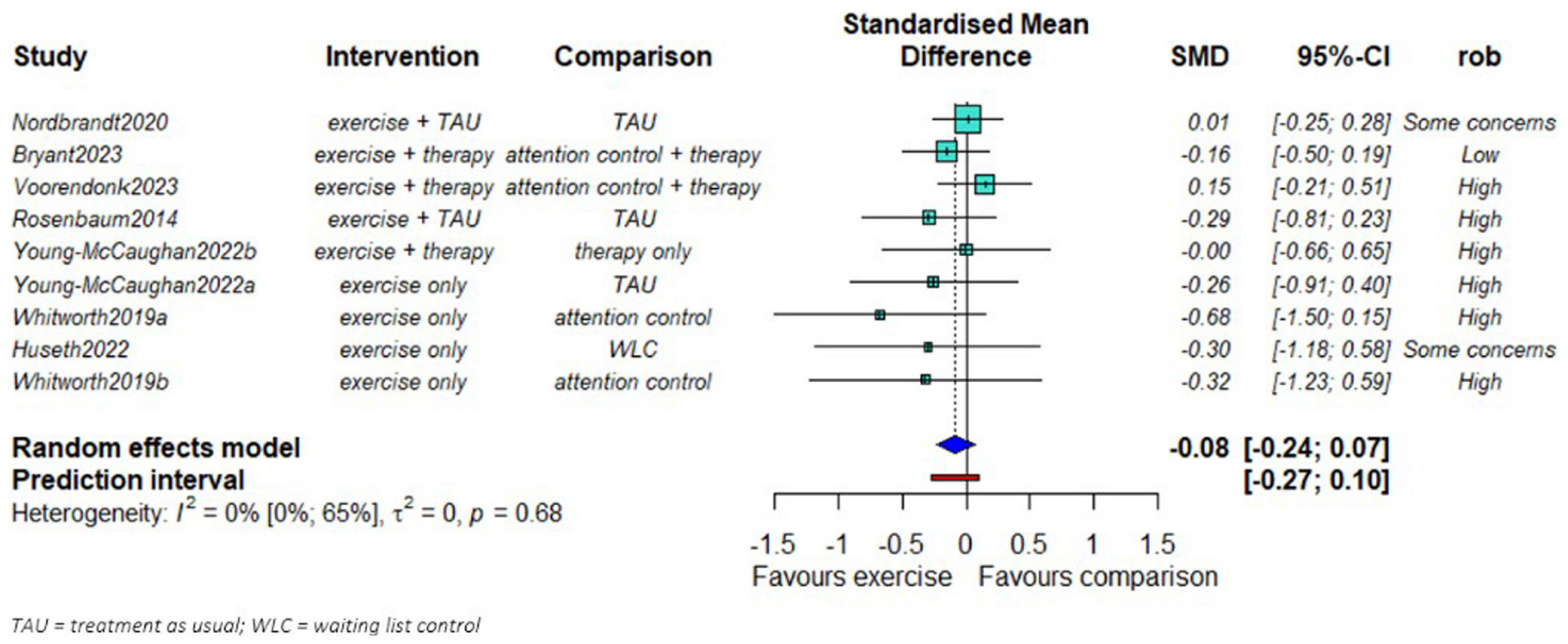
Mata-analysis of the effects of exercise on PTSD symptom severity (n = 655).

We found no evidence of a difference in PTSD symptom severity reduction between exercise and comparison groups (SMD = -0.08; 95% CI: -0.24 to 0.07, Figure 2.). No statistical heterogeneity was observed (τ ^2^=0). The smaller studies showed larger effects favoring the exercise groups compared to the larger studies.

We found some evidence that the effect of exercise might be larger in studies of high-intensity exercise compared to studies with moderate intensity ( Extended data). We did not find any important differences between the effects of aerobic, anaerobic, or mixed exercise groups ( Extended data). We found that studies with exercise alone as intervention were associated with larger effects than those in studies where patients were additionally given treatment-as-usual or psychotherapy ( Extended data).

The meta-regression analysis coefficient for exercise duration was 0.02 (95% CI -0.01 to 0.05; Extended data); for every additional week, the SMD in PTSD symptoms increases by 0.02 SDs. The SMD from the three studies with low risk of bias or some concerns, was -0.06, 95% CI -0.27 to 0.14 ( Extended data). However, as we had only 9 comparisons for these outcome, interpretation of results from meta-regression and subgroup analyses reported here should be done conservatively.

Visual inspection of the contour-enhanced funnel plot suggested that the preponderance of small studies with positive results in favour of exercise, could be due to publication bias ( Extended data).

### Secondary outcomes

The Extended data provides a synthesis of the secondary outcomes, for which there was insufficient data to conduct a meta-analysis (functional impairment, PTSD symptom clusters, and loss of PTSD diagnosis).

The forest plot for study dropout suggests no evidence of a difference between exercise and comparison groups (RR 1.28, 95% CI 0.67 to 2.45) but with large heterogeneity (95% PrI 0.16 to 10.15; Extended data).

### Evidence about mediation

The Extended data provides details of the three meditation studies at high risk of bias ^38, 40, 42^. In Crombie *et al.*, the exercise group exhibited lower threat expectancy ratings following reinstatement than the comparison group ( *p* = .032). In Powers *et al.* ^38^ the exercise increased BDNF concentration more than the control (Cohen's d = 1.08) and had a significantly greater reduction in PTSD symptom compared to control (Cohen's d = 2.65). In Whitworth *et al.* ^40^ changes in the perception of the resistance training sessions (cognitive appraisal; p = 0.02) and perceived exertion ( *p* = 0.01) mediated the relationship between exercise and PTSD symptom severity. Affect, arousal, and distress, were also examined as mediators with insufficient evidence about their role (p-values 0.63, 0.12 and 0.17 respectively).

### Findings from non-human studies

**Figure 3.**
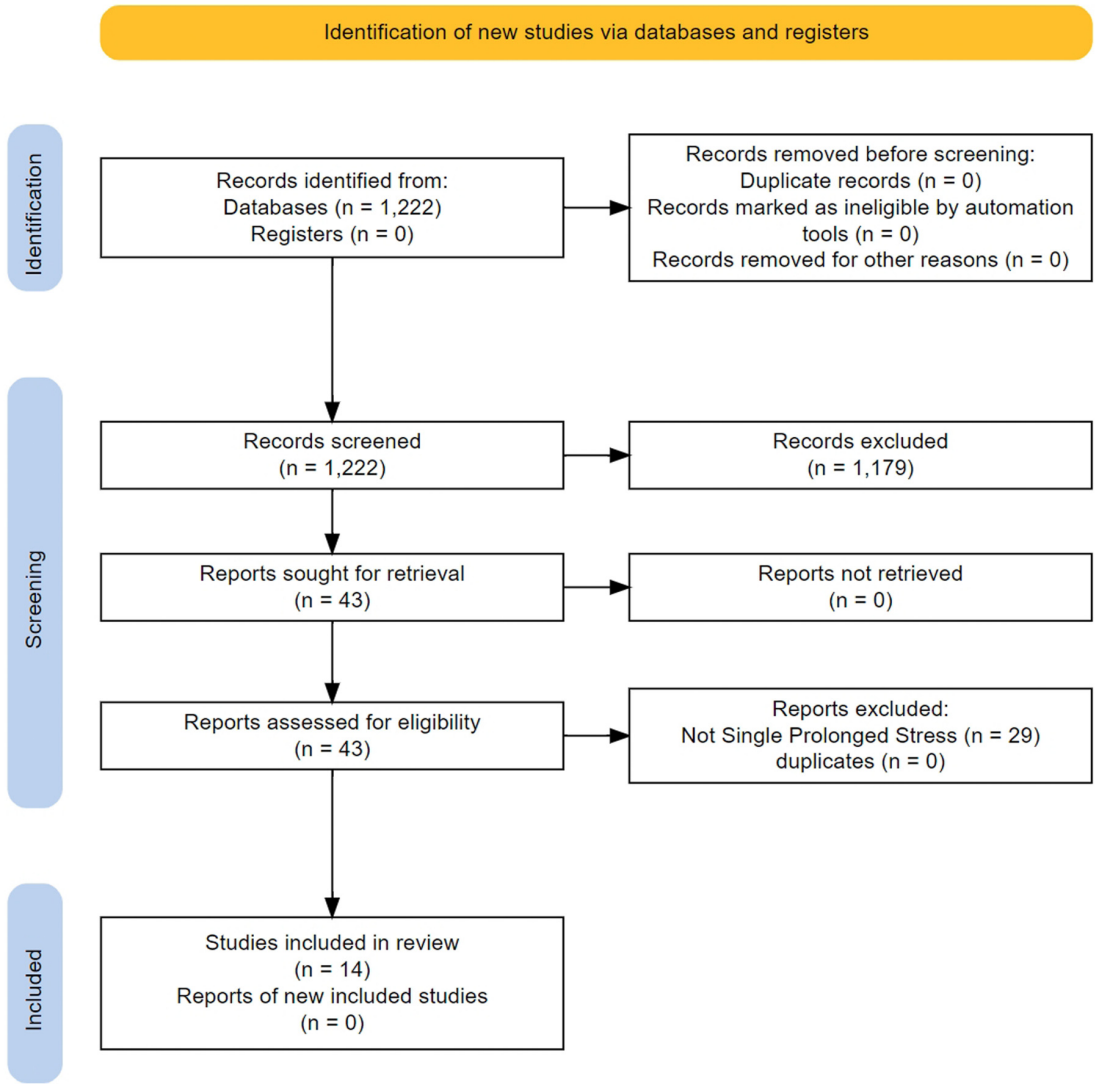
Flow of study selection and descriptives for the non-human studies.

We screened 1220 titles/abstracts and 43 full texts ( Figure 3). The main characteristics of the 14 studies that were finally included in the analysis are presented in Extended data.

### Behavioural outcomes

Considering all behavioural outcomes together, 74 experimental comparisons were reported in 19 experiments from 14 publications involving two animal strains and reporting data from 1192 animals. Multilevel meta-analysis showed an improvement in behaviour between exercise and control groups, with very large heterogeneity (SMD = 1.33, 95% CI 0.89 to 1.78; 95% PrI -0.24 to 2.90; Extended data).

We found no evidence that sex ( Extended data), voluntary vs forced exercise ( Extended data), the number of week of treatment ( Extended data), or exercise intensity ( Extended data), modifies substantially the effect of exercise.

We found an inverse relationship between total exercise in km (calculated as the product of the number of sessions, session duration, and session intensity) and behavioural outcomes, with a reduction in the beneficial effect of exercise by 0.06 SMD = units (95% CI 0.022 to 0.097) for every additional km of exercise ( Extended data).

Analysing each behavioural outcome separately, we found that exercise increased locomotor activity (SMD 1.30, 95% CI 0.74 to 1.87; 95% PrI -0.47 to 3.08; 13 comparisons in 4 publications; Extended data), improved fear memory (SMD = 2.55, 95% CI 1.29 to 3.82; 95% PrI -0.54 to 5.65; 7 comparisons in 4 publications Extended data), and improved freezing behaviour (SMD = 1.06, 95% CI 0.20 to 1.93; 95% PrI 0.18 to 1.94; 4 comparisons in 3 publications; Extended data). However, heterogeneity was generally substantial.

### Biological and neurological outcomes

Heterogeneity was large in most of the outcomes observed.

***BDNF levels***

We found that exercise was associated with increased BDNF levels (SMD = 1.79, 95% CI 0.56 to 3.01; 95% PrI -1.63 to 5.21; 24 comparisons in 11 experiments from 8 publications Extended data). The association was not modified by sex ( Extended data), voluntary vs forced exercise ( Extended data), or number of weeks of treatment ( Extended data). We found an inverse relationship between exercise intensity (in m/sec) with BDNF levels, with a reduction in SMD = 0.180 (95% CI 0.055 to 0.304) for every additional m/s speed ( Extended data). Further, we found a similar inverse relationship between total exercise and BDNF levels, with a reduction in BDNF levels of 0.077 (95% CI 0.024 to 0.129) for every additional km of total exercise ( Extended data).

***Biological stress response***

There was no significant effect of exercise on stress response (SMD = 2.03, 95% CI -1.78 to 5.84; 95% PrI -7.88 to 11.93; 10 comparisons in 8 experiments from 6 publications Extended data).

***Neurotransmitter levels and other neurobiological outcomes***

We found no evidence that exercise modifies the neurotransmitter levels (SMD = 0.07; 95% CI -0.32 to 0.46; 95% PrI -0.70 to 0.83; 10 comparisons in 1 publication Extended data).

Analysing together all neurobiological outcomes, we found no evidence of association with exercise (SMD = 1.10, 95% CI -0.18 to 2.38; 95% PrI -2.79 to 4.99; 62 experimental comparisons in 11 experiments from 9 publications; Extended data).

### Post-hoc analyses of the outcomes from non-human studies

To provide context, the Extended data shows how SPS was associated with changes in neurobiological and behavioural outcomes compared with control groups not exposed to SPS. In summary, we found evidence that SPS is associated with worsening of behavioural outcomes (including reduced locomotor activity and increased fear memory) but had no significant effects on neurobiological outcomes (including BDNF and neurotransmitter levels). We estimated correlations between different outcome measures, in cases where different outcomes were measured in the same cohorts of animals. Surprisingly, given the absence of a significant effect of SPS on BDNF levels, we found that effects sizes for neurobiological outcomes had a stronger relationship with behavioural outcomes in the modelling of SPS (r ^2^ = .37) than in the effect of exercise on the SPS phenotype (r ^2^ = 0.07). We found strong correlations between stress response, BDNF levels, and other neurobiological outcomes in both modelling and exercise, and for BDNF and for stress responses with behavioural outcomes.

### Sensitivity analyses

Different assumptions for the imputed within-study correlation and the use of NMD rather than SMD effect size estimates, gave different magnitudes of effect but did not materially alter the direction of effect or the total interpretation of the summary effects ( Extended data).

### Small-study effects

There was no evidence for small-study effects for any of the outcomes measured ( Extended data).

## Discussion

This review aimed to synthesize evidence from both human and non-human studies on how exercise impacts PTSD symptom severity and functional impairment. While prior meta-analyses suggested that exercise could reduce PTSD symptoms, our findings from the meta-analysis of human studies did not support this. Although six out of nine studies showed more favorable outcomes in the exercise group, three larger studies reported no significant effect, suggesting that study size may influence the observed results. Despite this, some evidence from single studies indicated that exercise might improve functional impairment. Notably, findings from non-human studies strongly supported the benefits of exercise for PTSD.

 Contrary to our findings, earlier meta-analyses reported significantly greater improvement in PTSD symptom severity for participants in exercise compared to comparison groups post-intervention. However, these earlier studies exhibited considerable heterogeneity in design, participants, interventions, and outcomes, making their results difficult to generalize. For example, a recent meta-analysis reported small to medium effects of exercise on PTSD symptoms in RCTs, while another highlighted similar effects across mind-body exercises, including yoga and relaxation practices ^43^. These meta-analyses incorporated mindfulness-based elements into exercise interventions, complicating the isolation of exercise’s standalone effects.

To elucidate the effects of exercise on PTSD symptom severity, we examined putative moderating variables.

In human studies, we found some evidence suggesting a difference between the effects of moderate-intensity and high-intensity exercise on PTSD symptom severity. Specifically, high-intensity exercise appears to be more effective in reducing PTSD symptoms compared to moderate-intensity exercise. Other human research suggests that high-intensity exercise is more effective than moderate-intensity exercise in reducing PTSD symptoms in healthy adults ^44^.

High-intensity exercise reduces PTSD symptoms and increases BDNF levels, suggesting that BDNF may mediate the beneficial effects of exercise on PTSD-related outcomes ^38, 39^. Other human research has found that high-intensity exercise significantly increases BDNF levels compared to low and moderate-intensity exercise ^45^. This effect is particularly pronounced in men with lower physical fitness levels ^45^. In contrast, another study found no significant difference in BDNF response between healthy men undergoing high-intensity, low-volume resistance training and those undergoing low-intensity, high-volume resistance training ^46^.

Compared to human studies, findings from non-human studies are mixed. Higher exercise intensity was associated with increased stress levels and a reduction in BDNF levels. Moreover, while higher exercise intensity (measured in meters per second) is positively related to certain neurobiological markers (excluding BDNF), it does not appear to affect behavioral outcomes.

Our review found no significant differences in PTSD symptom outcomes between studies that used exercise alone and those that combined it with psychotherapy or treatment-as-usual. However, visual inspection of forest plots suggested that studies focusing solely on exercise interventions had larger effect sizes than those combining exercise with other treatments. This highlights the potential efficacy of exercise as a standalone intervention for PTSD, though more research is needed to clarify its role relative to other treatment modalities. In the current study, dropout rates revealed no notable difference in dropout between the exercise and comparison groups. While initial observations suggested a potentially higher dropout in the comparison groups based on confidence levels, closer examination of the forest plot suggests that this discrepancy may be primarily attributed to a single study with a large sample and a high number of dropouts.

Increase in circulating levels of BDNF was found to mediate the effects of exercise in reducing PTSD symptoms. Indirectly, increasing BDNF during aerobic exercise was found to significantly reduce threat expectancy ratings following reinstatement This aligns with previous research showing elevated BDNF levels in individuals with PTSD, which may facilitate a greater response to treatment. Exercise-induced increases in BDNF may thus contribute to its neuroprotective effects, enhancing cognitive and emotional resilience in PTSD patients.

AEA, an endocannabinoid, increases during exercise and helps reduce threat expectancy following reinstatement. While earlier studies reported conflicting findings on AEA levels in PTSD patients ^47, 48^, this review found that moderate-intensity aerobic exercise can lower threat perceptions by mediating AEA levels ^39^. This suggests that AEA, an endocannabinoid, may play a role in the psychological benefits of aerobic exercise, particularly in reducing anxiety or threat perceptions. AEA may enhance the psychological benefits of exercise by improving fear extinction memory, reducing stress reactivity, and protecting against negative emotional responses linked to PTSD.

Comparing human and non-human studies reveals interesting discrepancies. While non-human studies consistently showed improvements in PTSD-like symptoms after exercise, human studies produced more variable results. One explanation could be the greater control over experimental conditions in non-human studies, such as the induction of PTSD-like phenotypes and the administration of exercise interventions. Additionally, reduced heterogeneity in non-human studies likely contributed to larger effect size estimates.

A crucial distinction between human and non-human studies involves the interpretation of physical activity. Increased locomotor activity in animal models is often considered a sign of reduced anxiety, whereas in humans, elevated activity could be interpreted as psychomotor agitation, a symptom of PTSD itself. These nuances complicate direct comparisons but offer valuable insights into how exercise might differentially impact human and animal populations.

### Strengths

One of the main strengths of this review is its focus on exercise without the confounding effects of mindfulness-based practices. This allowed for a clearer examination of the specific effects of physical activity on PTSD. Additionally, this review is among the first to explore the biological and psychosocial mechanisms underlying exercise’s therapeutic effects on PTSD, highlighting the scarcity of mechanistic studies in this area.

### Limitations

However, several limitations should be noted. Due to the limited number of mechanistic studies available, we were unable to examine these mechanisms in depth. Furthermore, the small number of included RCTs limits the generalizability of our findings. The review was also restricted to English-language studies, and few of the included studies reported long-term outcomes. Only three studies examined potential mediators of exercise’s effects, and none provided data for PTSD symptom severity post-intervention, which precluded a meta-analysis of mediator effects. Additionally, the small sample sizes and lack of rigorous reporting in the non-human studies limited the clarity of their findings.

## Future recommendations

Future research should investigate potential moderators and mediators of the effects of exercise on PTSD, including variables like exercise type, intensity, duration, and timing. These factors might explain some of the variance in study outcomes. Additionally, more studies are needed to explore the potential benefits of high-intensity exercise, which showed promising but inconclusive trends in this review.

Moreover, future trials should aim to disentangle the effects of exercise from other therapeutic elements, such as mindfulness, and explore how exercise interventions can be tailored to maximize their benefits for PTSD. Given the mixed results of combining exercise with psychotherapy, sophisticated trial designs should assess treatment effect heterogeneity and mediational pathways to provide a stronger foundation for personalized therapies for PTSD.

## Conclusions

While exercise did not significantly reduce PTSD symptom severity across studies, its potential to improve functional outcomes and mediate neurobiological pathways like BDNF and AEA suggests that it remains a promising therapeutic avenue. Future studies should focus on understanding the differential effects and mechanisms of exercise interventions and their potential as standalone or adjunctive treatments for PTSD.

## Ethics and consent

Ethical approval and consent were not required.

## Data Availability

The data for this article consists of bibliographic references, which are included in the References section. The archived aggregated data and analysis code at time of publication are also in the GALENOS data repository: Human studies: Analysis code available from: https://github.com/galenos-project/LSR2_exercise_H/ Archived analysis code at time of publication: https://doi.org/10.60818/g2scz-79s60 ^49^ Non-human studies: Analysis code available from: https://github.com/galenos-project/LSR2_exercise_A Archived analysis code at time of publication: https://doi.org/10.60818/y76xf-xz514 ^50^ Underlying data are available under the terms of the Creative Commons Attribution 4.0 International license (CC-BY 4.0). Open Science Framework: The therapeutic potential of exercise in post-traumatic stress disorder and its underlying mechanisms: A living systematic review of human and non-human studies. https://doi.org/10.17605/OSF.IO/Q8UHZ ^51^. The project contains the following extended data: Open Science Framework: Extended data: 1st iteration 2024: Detailed report of the human studies. https://osf.io/v2sqn Open Science Framework: Extended data: 1st iteration 2024: Detailed report of non-human studies. https://osf.io/2usxc Extended data are available under the terms of the Creative Commons Attribution 4.0 International license (CC-BY 4.0). Open Science Framework: PRISMA checklist for ‘The therapeutic potential of exercise in post-traumatic stress disorder and its underlying mechanisms: A living systematic review of human and non-human studies’. https://doi.org/10.17605/OSF.IO/Q8UHZ ^51^. Completed checklists for the corresponding reporting guidelines are available under the terms of the Creative Commons Attribution 4.0 International license (CC-BY 4.0).

## References

[1] American Psychiatric Association: Diagnostic and Statistical Manual of Mental Disorders.5th ed. Arlington, VA, USA: American Psychiatric Publishing,2013. Reference Source

[2] Watkins LE Sprang K Rothbaum BO : Treating PTSD: a review of evidence-based psychotherapy interventions. *Front Behav Neurosci.* 2018;12:258. 10.3389/fnbeh.2018.00258 30450043 PMC6224348

[3] Bisson JI Berliner L Cloitre M : The international society for traumatic stress studies new guidelines for the prevention and treatment of posttraumatic stress disorder: methodology and development process. *J Trauma Stress.* 2019;32(4):475–83. 10.1002/jts.22421 31283056

[4] Weisman JS Rodebaugh TL : Exposure therapy augmentation: a review and extension of techniques informed by an inhibitory learning approach. *Clin Psychol Rev.* 2018;59:41–51. 10.1016/j.cpr.2017.10.010 29128146

[5] Foa EB Gillihan SJ Bryant RA : Challenges and successes in dissemination of evidence-based treatments for posttraumatic stress: lessons learned from prolonged exposure therapy for PTSD. *Psychol Sci Public Interest.* 2013;14(2):65–111. 10.1177/1529100612468841 25722657 PMC4338436

[6] Caspersen CJ Powell KE Christenson GM : Physical activity, exercise, and physical fitness: definitions and distinctions for health-related research. *Public Health Rep.* 1985;100(2):126–31. 3920711 PMC1424733

[7] Rosenbaum S Sherrington C Tiedemann A : Exercise augmentation compared with usual care for post-traumatic stress disorder: a randomized controlled trial. *Acta Psychiatr Scand.* 2015;131(5):350–9. 10.1111/acps.12371 25443996

[8] Noetel M Sanders T Gallardo-Gómez D : Effect of exercise for depression: systematic review and network meta-analysis of randomised controlled trials. *BMJ.* 2024;384: e075847. 10.1136/bmj-2023-075847 38355154 PMC10870815

[9] Basso JC Suzuki WA : The effects of acute exercise on mood, cognition, neurophysiology, and neurochemical pathways: a review. *Brain Plast.* 2017;2(2):127–52. 10.3233/BPL-160040 29765853 PMC5928534

[10] Winters BL Vaughan CW : Mechanisms of endocannabinoid control of synaptic plasticity. *Neuropharmacology.* 2021;197: 108736. 10.1016/j.neuropharm.2021.108736 34343612

[11] Jay TM : Dopamine: a potential substrate for synaptic plasticity and memory mechanisms. *Prog Neurobiol.* 2003;69(6):375–90. 10.1016/s0301-0082(03)00085-6 12880632

[12] Santini E Huynh TN Klann E : Mechanisms of translation control underlying long-lasting synaptic plasticity and the consolidation of long-term memory. *Prog Mol Biol Transl Sci.* 2014;122:131–67. 10.1016/B978-0-12-420170-5.00005-2 24484700 PMC6019682

[13] Kowiański P Lietzau G Czuba E : BDNF: a key factor with multipotent impact on brain signaling and synaptic plasticity. *Cell Mol Neurobiol.* 2018;38(3):579–93. 10.1007/s10571-017-0510-4 28623429 PMC5835061

[14] Pitts BL Whealin JM Harpaz-Rotem I : BDNF Val66Met polymorphism and posttraumatic stress symptoms in U.S. military veterans: protective effect of physical exercise. *Psychoneuroendocrinology.* 2019;100:198–202. 10.1016/j.psyneuen.2018.10.011 30388593

[15] Peters JL Sutton AJ Jones DR : Contour-enhanced meta-analysis funnel plots help distinguish publication bias from other causes of asymmetry. *J Clin Epidemiol.* 2008;61(10):991–6. 10.1016/j.jclinepi.2007.11.010 18538991

[16] Motta R : The role of exercise in reducing PTSD and negative emotional states.2018. 10.5772/intechopen.81012

[17] Price M Lancaster CL Gros DF : An examination of social support and PTSD treatment response during prolonged exposure. *Psychiatry.* 2018;81(3):258–70. 10.1080/00332747.2017.1402569 30020026 PMC6207452

[18] Zalta AK Tirone V Orlowska D : Examining moderators of the relationship between social support and self-reported PTSD symptoms: a meta-analysis. *Psychol Bull.* 2021;147(1):33–54. 10.1037/bul0000316 33271023 PMC8101258

[19] Mirjalili R Shokouh E Dehkordi NS : Prior short-term exercise prevents behavioral and biochemical abnormalities induced by single prolonged stress in a rat model of posttraumatic stress disorder. *Behav Brain Res.* 2022;428: 113864. 10.1016/j.bbr.2022.113864 35405172

[20] Borghans B Homberg JR : Animal models for Post-Traumatic Stress Disorder: an overview of what is used in research. *World J Psychiatry.* 2015;5(4):387–96. 10.5498/wjp.v5.i4.387 26740930 PMC4694552

[21] Verbitsky A Dopfel D Zhang N : Rodent models of Post-Traumatic Stress Disorder: behavioral assessment. *Transl Psychiatry.* 2020;10(1): 132. 10.1038/s41398-020-0806-x 32376819 PMC7203017

[22] Wu Z Tian Q Li F : Behavioral changes over time in Post-Traumatic Stress Disorder: insights from a rat model of single prolonged stress. *Behav Processes.* 2016;124:123–9. 10.1016/j.beproc.2016.01.001 26772783

[23] Schöner J Heinz A Endres M : Post-Traumatic Stress Disorder and beyond: an overview of rodent stress models. *J Cell Mol Med.* 2017;21(10):2248–56. 10.1111/jcmm.13161 28374949 PMC5618668

[24] Cipriani A Seedat S Milligan L : New living evidence resource of human and non-human studies for early intervention and research prioritisation in anxiety, depression and psychosis. *BMJ Ment Health.* 2023;26(1): e300759. 10.1136/bmjment-2023-300759 37290906 PMC10255027

[25] Chiocchia V Salanti G Siafis S : Protocol for living systematic reviews in GALENOS research programme: a generic template for systematic reviews aiming to evaluate associations.2023. 10.17605/OSF.IO/ZN5P4

[26] Wright S Furukawa T Macleod M : Mechanisms through which exercise reduces symptom severity and/or functional impairment in Posttraumatic Stress Disorder (PTSD): protocol for a living systematic review of human and non-human studies [version 1; peer review: 1 approved with reservations]. *Wellcome Open Res.* 2023;8:494. 10.12688/wellcomeopenres.19903.1 PMC12102654 40416511

[27] Page MJ McKenzie JE Bossuyt PM : The PRISMA 2020 statement: an updated guideline for reporting systematic reviews. *BMJ.* 2021;372:n71. 10.1136/bmj.n71 33782057 PMC8005924

[28] EPPI reviewer.2023. Reference Source

[29] Bahor Z Liao J Currie G : Development and uptake of an online systematic review platform: the early years of the CAMARADES Systematic Review Facility (SyRF). *BMJ Open Sci.* 2021;5(1): e100103. 10.1136/bmjos-2020-100103 35047698 PMC8647599

[30] Higgins JPT Savović J Page MJ : Cochrane handbook for systematic reviews of interventions version.In: Higgins JPT, Thomas J, Chandler J, Cumpston M, Li T, Page MJ, *et al.*, editors. *Assessing risk of bias in a randomized trial* . Cochrane Training;2022.

[31] Murillo C Vo TT Vansteelandt S : How do psychologically based interventions for chronic musculoskeletal pain work? A systematic review and meta-analysis of specific moderators and mediators of treatment. *Clin Psychol Rev.* 2022;94: 102160. 10.1016/j.cpr.2022.102160 35561510 PMC11146991

[32] Hooijmans CR Rovers MM de Vries RB : SYRCLE’s risk of bias tool for animal studies. *BMC Med Res Methodol.* 2014;14(1): 43. 10.1186/1471-2288-14-43 24667063 PMC4230647

[33] Percie du Sert N Hurst V Ahluwalia A : The ARRIVE guidelines 2.0: updated guidelines for reporting animal research. *PLoS Biol.* 2020;18(7): e3000410. 10.1371/journal.pbio.3000410 32663219 PMC7360023

[34] Cashin AG McAuley JH Lamb SE : Development of A Guideline for Reporting Mediation Analyses (AGReMA). *BMC Med Res Methodol.* 2020;20(1): 19. 10.1186/s12874-020-0915-5 32013883 PMC6998151

[35] Yang Y Macleod M Pan J : Advanced methods and implementations for the meta-analyses of animal models: current practices and future recommendations. *Neurosci Biobehav Rev.* 2023;146: 105016. 10.1016/j.neubiorev.2022.105016 36566804

[36] Campbell M McKenzie JE Sowden A : Synthesis Without Meta-analysis (SWiM) in systematic reviews: reporting guideline. *BMJ.* 2020;368: l6890. 10.1136/bmj.l6890 31948937 PMC7190266

[37] Greene DR Petruzzello SJ : Working it out: acute exercise to combat anxiety and depressive symptoms in individuals living with subsyndromal Post-Traumatic Stress Disorder. *Int J Sport Exerc Psychol.* 2022;20(5):1416–31. 10.1080/1612197X.2021.1979075

[38] Powers MB Medina JL Burns S : Exercise augmentation of exposure therapy for PTSD: rationale and pilot efficacy data. *Cogn Behav Ther.* 2015;44(4):314–27. 10.1080/16506073.2015.1012740 25706090 PMC4464974

[39] Crombie KM Sartin-Tarm A Sellnow K : Exercise-induced increases in anandamide and BDNF during extinction consolidation contribute to reduced threat following reinstatement: preliminary evidence from a randomized controlled trial. *Psychoneuroendocrinology.* 2021;132: 105355. 10.1016/j.psyneuen.2021.105355 34280820 PMC8487992

[40] Whitworth JW Nosrat S SantaBarbara NJ : Feasibility of resistance exercise for Posttraumatic Stress and Anxiety Symptoms: a randomized controlled pilot study. *J Trauma Stress.* 2019a;32(6):977–84. 10.1002/jts.22464 31743507

[41] Young-McCaughan S Peterson AL Mintz J : Testing the role of aerobic exercise in the treatment of Posttraumatic Stress Disorder (PTSD) symptoms in U.S. active duty military personnel: a pilot study. *Cogn Behav Ther.* 2022;51(4):309–25. 10.1080/16506073.2021.2001689 35001842

[42] Crombie KM Sartin-Tarm A Sellnow K : Aerobic exercise and consolidation of fear extinction learning among women with Posttraumatic Stress Disorder. *Behav Res Ther.* 2021a;142: 103867. 10.1016/j.brat.2021.103867 34020153

[43] van de Kamp MM Scheffers M Emck C : Body-and movement-oriented interventions for Posttraumatic Stress Disorder: an updated systematic review and meta-analysis. *J Trauma Stress.* 2023;36(5):835–48. 10.1002/jts.22968 37702005

[44] Borrega-Mouquinho Y Sánchez-Gómez J Fuentes-García JP : Effects of High-Intensity Interval Training and Moderate-Intensity Training on stress, depression, anxiety, and resilience in healthy adults during coronavirus disease 2019 confinement: a randomized controlled trial. *Front Psychol.* 2021;12: 643069. 10.3389/fpsyg.2021.643069 33716913 PMC7943442

[45] Antunes BM Rossi FE Teixeira AM : Short-time high-intensity exercise increases peripheral BDNF in a physical fitness-dependent way in healthy men. *Eur J Sport Sci.* 2020;20(1):43–50. 10.1080/17461391.2019.1611929 31057094

[46] Dinoff A Herrmann N Swardfager W : The effect of exercise training on resting concentrations of peripheral Brain-Derived Neurotrophic Factor (BDNF): a meta-analysis. *PLoS One.* 2016;11(9): e0163037. 10.1371/journal.pone.0163037 27658238 PMC5033477

[47] Hauer D Schelling G Gola H : Plasma concentrations of endocannabinoids and related primary fatty acid amides in patients with Post-Traumatic Stress Disorder. *PLoS One.* 2013;8(5): e62741. 10.1371/journal.pone.0062741 23667516 PMC3647054

[48] Amatriain-Fernández S Budde H Gronwald T : The endocannabinoid system as modulator of exercise benefits in mental health. *Curr Neuropharmacol.* 2021;19(8):1304–22. 10.2174/1570159X19666201218112748 33342414 PMC8719298

[49] Wright S : LSR2 Exercise for PTSD - Human Studies.(Version 1). [Data set], GALENOS. 2024. 10.60818/g2scz-79s60

[50] Macleod M : LSR2 Exercise for PTSD - Non-human Studies.(Version 1). [Data set], GALENOS. 2024. 10.60818/y76xf-xz514

[51] Wright S Salanti G Chiocchia V : The therapeutic potential of exercise in Post-Traumatic Stress Disorder and its underlying mechanisms: a living systematic review of human and non-human studies.November 4,2024. 10.17605/OSF.IO/Q8UHZ PMC11959255 40171151

