# Supplementary material for: The therapeutic potential of exercise in post-traumatic stress disorder and its underlying mechanisms: A living systematic review of human and non-human studies
Source: Wellcome Open Res. 2025 May 27;9:720. Originally published 2024 Dec 7. [Version 4] doi: 10.12688/wellcomeopenres.23033.4 (PMC11959255; doi:10.12688/wellcomeopenres.23033.4)
Supplement: Peer review history

# Reviewer report: Reviewer response for version 2

**DOI:** 10.21956/wellcomeopenres.26150.r115723

**Published:** 2025-03-31

**Authors:** Jony Sheynin

**Affiliations:** 1 Texas A&M University, Houston, Texas, USA

I appreciate the invitation to review this interesting manuscript. Indeed, it is important to understand the evidence from human and non-human literature on the therapeutic potential and the underlying mechanism of exercise in PTSD. Such knowledge would have a direct relevance to treatment of PTSD, and could also have implications for other mental conditions. Below I list some suggestions that authors might want to consider to further improve the manuscript:

- Abstract: “Results”: Include results from “non-human studies”, as well.
- As the concept of a “living review” is relatively novel, it would be helpful to define it at the beginning of the manuscript.
- Methods; “Eligibility criteria” - “participants with either above-threshold symptoms on any standardized self-report measure or a clinical diagnosis of PTSD.”: “Future recommendations” could include studying participants with sub-threshold PTSD symptoms, i.e., individuals who develop some symptoms but are not diagnosed with PTSD, per se. Along the same line, there is evidence that trauma alone could cause alterations in neural signal (Lokshina et al 2021, Current Research in Psychiatry); Could exercise have positive effect then, as well?
- Methods; “Eligibility criteria”: Please clarify why only the SPS animal model of PTSD has been included in the review of non-human studies (see Richter-Levin et al. 2019, PMID: 30816289).
- Methods; “Outcomes”: “For human studies, the primary outcome was PTSD symptom severity....”: please clarify that this refers to *total* symptoms, whereas the secondary outcomes refer to *symptom clusters*.
- Methods; “Study identification”: It’s not clear why search strategies for human and non-human studies used different databases; For instance, Why PubMed and preprints were used only for non-human studies?
- It would be helpful to clarify in the main text (i.e., not only Table 1) what are the comparison groups/sham interventions that have been reported in the studies.
- Results; “PTSD symptom severity”: “We found that studies with exercise alone as intervention were associated with larger effects than those in studies where patients were additionally given treatment-as-usual or psychotherapy.”: This is misleading. Please first clarify that there were no significant differences. Also, edit “effects” to “effect sizes”. Lastly, this finding is somewhat counter-intuitive; Why do authors think that the additional “treatment-as-usual” might reduce the positive effect of exercise?
- There is growing literature on the neural biomarkers involved in PTSD treatment, as detected by functional MRI (e.g., see Rauch et al. 2018, PMID: 29081351). However, in the current manuscript the research on neuroimaging/fMRI is not discussed. I suggest to elaborate on this topic, and possibly propose studying this in the “Future recommendations” section.
- The manuscript mentions the positive effect of BDNF and AEA on fear extinction in PTSD; It would be helpful to first, mention the evidence on altered fear extinction in PTSD (see Lokshina et al. 2023, PMID: 37535308).
- Authors mention that exercise-induced increase in BDNF may increase cognitive and emotional resilience in PTSD patients; Do authors think that indeed exercise could help with developing resilience to the development of PTSD? Could this be investigated in future work in individual before the occurrence of a traumatic event (e.g., in pre-deployment soldiers).

Are the rationale for, and objectives of, the Systematic Review clearly stated?

Yes

Is the statistical analysis and its interpretation appropriate?

Yes

If this is a Living Systematic Review, is the ‘living’ method appropriate and is the search schedule clearly defined and justified? (‘Living Systematic Review’ or a variation of this term should be included in the title.)

Yes

Are sufficient details of the methods and analysis provided to allow replication by others?

Yes

Are the conclusions drawn adequately supported by the results presented in the review?

Yes

Reviewer Expertise:

PTSD, trauma, neuroimaging, fMRI, neural activation, neural connectivity, computational psychiatry

I confirm that I have read this submission and believe that I have an appropriate level of expertise to confirm that it is of an acceptable scientific standard, however I have significant reservations, as outlined above.

## References

[1] : Integrating biological treatment mechanisms into randomized clinical trials: Design of PROGrESS (PROlonGed ExpoSure and Sertraline Trial). *Contemp Clin Trials* .2018;64: 10.1016/j.cct.2017.10.013 128-138 10.1016/j.cct.2017.10.013 29081351

[2] : Fear Extinction Learning in Posttraumatic Stress Disorder. *Curr Top Behav Neurosci* .2023;64: 10.1007/7854_2023_436 257-270 10.1007/7854_2023_436 37535308

[3] : Animal models of PTSD: a challenge to be met. *Mol Psychiatry* .2019;24(8) : 10.1038/s41380-018-0272-5 1135-1156 10.1038/s41380-018-0272-5 30816289 PMC6756084

[4] Lokshina et al 2021, Current Research in Psychiatry; https://doi.org/10.46439/Psychiatry.1.015.

# Reviewer report: Reviewer response for version 2

**DOI:** 10.21956/wellcomeopenres.26150.r118167

**Published:** 2025-02-06

**Authors:** Ryan S Garten

**Affiliations:** 1 Virginia Commonwealth University, Richmond, Virginia, USA

Thank you to the authors for addressing my comments. The changes made by the authors are adequate and have no further comments

Are the rationale for, and objectives of, the Systematic Review clearly stated?

Yes

Is the statistical analysis and its interpretation appropriate?

Yes

If this is a Living Systematic Review, is the ‘living’ method appropriate and is the search schedule clearly defined and justified? (‘Living Systematic Review’ or a variation of this term should be included in the title.)

Yes

Are sufficient details of the methods and analysis provided to allow replication by others?

Yes

Are the conclusions drawn adequately supported by the results presented in the review?

Yes

Reviewer Expertise:

My expertise is in exercise physiology with a focus on areas such as cardiovascular physiology and redox balance.

I confirm that I have read this submission and believe that I have an appropriate level of expertise to confirm that it is of an acceptable scientific standard.

# Reviewer report: Reviewer response for version 1

**DOI:** 10.21956/wellcomeopenres.25365.r114973

**Published:** 2025-01-30

**Authors:** Dharani Keyan

**Affiliations:** 1 School of Psychology, University of New South Wales, Sydney, Australia

**Summary of manuscript:**

 This paper reports on a living systematic review of the use of exercise as a potential adjunct for psychotherapy for PTSD. A living systematic review is justified within the context that the study of exercise within the field of human and non-human emotional memories has seen a burgeoning focus over the past decade. The manuscript is presented in three parts. The first part briefly reviews the potential biological and psychosocial mechanisms underpinning exercise induced benefits, with some focus on extinction related processes. Human studies comprise largely of treatment trials with the focal outcome as PTSD symptom severity. Non-human studies are limited to those using the single prolonged stress model as a paradigm for modelling analogue PTSD symptoms. The second part presents the results of the systematic and meta-analytic review. Included human studies largely do not specifically explore mechanisms underpinning exercise induced benefits.  In comparison, non-human studies that are included in the review explore several different biological mechanisms likely underpinning exercise-induced benefits on behavioural outcomes indicative of PTSD symptomology including BDNF, neurotransmitters and stress. The third part attempts to make recommendations for how to approach understanding of biopsychosocial mechanisms underpinning exercise induced benefits for PTSD symptom reduction based on earlier sections of the manuscript.

**Review comments for consideration:**

 The manuscript does not fully meet the stated objectives, particularly regarding the exploration of the potential for exercise and related biopsychosocial mechanisms in modulating PTSD symptom severity. The exclusion of certain human and animal studies leaves significant gaps in the review. For example, human analogue studies (e.g., trauma film paradigms, fear conditioning, and extinction paradigms) that explore the role of exercise in emotional memory modulation that underpin the PTSD condition are notably absent. Additionally, the decision to limit animal studies to those using the SPS model is restrictive, and the assumption that other paradigms are irrelevant should be reconsidered.

 The current structure and focus of the manuscript do not adequately address the complexity of the role exercise plays in modulating PTSD symptoms across human and non-human models. Recent reviews have highlighted various biological mechanisms, but these are only briefly mentioned in the manuscript. The discussion of exercise’s potential to modulate PTSD symptoms—specifically, how it affects emotional memory processes—should be more nuanced.

**Recommendations for Revision:**

1. **Expand and critically appraise the extant literature**: An appropriate revision of the first part of this manuscript would begin with a thorough description of all potential paradigms that have investigated the role of exercise on the PTSD condition including human treatment trials, human analogue experiments, and non-human studies. It would then describe all potential mechanisms under consideration for exercise induced modulation of the PTSD condition. The potential for exercise in modulating human and non-human emotional memories is complex, and recent reviews have outlined mechanisms and interactions (i.e., Keyan D *et al*.(2019); Loprinzi P *et al*. (2019). Consider the extant literature to this end. The manuscript currently mentions the distraction hypothesis but does not engage with competing hypotheses. For instance, immediate exercise following analogue trauma exposure could potentially increase intrusive memories rather than reduce them. A more thoughtful consideration of alternative mechanisms is warranted, especially in light of studies such as Keyan D *et al*. (2017)
2. **Conduct an expanded literature search**: A broader literature search is recommended to capture all relevant empirical studies examining the role of exercise on human and non-human emotional memories analogous of PTSD symptomology. This should include a search of reference lists from all target studies and systematic and meta-analytic reviews that were populated from the searches. Since August 2023 (the last search update of this manuscript), several empirical studies on this subject have been published. The methods section should outline the update schedule for this review.  It would be useful for the reader to see the included studies listed in the reference list (perhaps with an * to indicate this).
3. **Clarify the state of the evidence in the results section:** The results section should present findings from human clinical trials, human analogue studies and non-human studies in a manner that allows independent scholars to interpret the evidence clearly. You might consider presenting graphical representations of the three different meta-analyses in the main sections of your paper. Without the inclusion of other animal paradigms (besides SPS) and human analogue studies, it is difficult to draw reliable conclusions about the state of evidence regarding the potential for exercise in the modulation processes that underpin the PTSD condition. An expansive search that does not presume that alternate animal paradigms yield irrelevant results but rather empirically tests whether study design characteristics are associated with different results, is preferred. This expanded focus is needed to adequately support the manuscript's conclusions.
4. **Discussion and conclusions**: Given the gap in the current manuscript, the discussion section should be expanded to better address potential discrepancies between human and non-human studies. The discussion should also explore how different doses and timings of exercise might influence the formation, modulation and resolution of emotional memories that underpin the PTSD condition.

Expanding the scope of the review, incorporating a more detailed discussion of mechanisms, and conducting a broader literature search will improve the quality and comprehensiveness of the manuscript. A revised version should more fully address the complexity of exercise’s potential role in modulating PTSD symptoms.

Are the rationale for, and objectives of, the Systematic Review clearly stated?

Yes

Is the statistical analysis and its interpretation appropriate?

Partly

If this is a Living Systematic Review, is the ‘living’ method appropriate and is the search schedule clearly defined and justified? (‘Living Systematic Review’ or a variation of this term should be included in the title.)

Partly

Are sufficient details of the methods and analysis provided to allow replication by others?

Yes

Are the conclusions drawn adequately supported by the results presented in the review?

No

Reviewer Expertise:

NA

I confirm that I have read this submission and believe that I have an appropriate level of expertise to confirm that it is of an acceptable scientific standard, however I have significant reservations, as outlined above.

## References

[1] : The capacity for acute exercise to modulate emotional memories: A review of findings and mechanisms. *Neurosci Biobehav Rev* .2019;107: 10.1016/j.neubiorev.2019.09.033 438-449 10.1016/j.neubiorev.2019.09.033 31562922

[2] : Exercise and Emotional Memory: a Systematic Review. *Journal of Cognitive Enhancement* .2019;3(1) : 10.1007/s41465-018-0086-z 94-103 10.1007/s41465-018-0086-z

[3] : Brief exercise enhances intrusive memories of traumatic stimuli. *Neurobiol Learn Mem* .2017;141: 10.1016/j.nlm.2017.03.012 9-13 10.1016/j.nlm.2017.03.012 28323200

# Response

**Published:** 2025-03-11

**Authors:** Simonne Wright87

**Affiliations:** Department of Psychiatry, Stellenbosch University, Stellenbosch, Western Cape, South Africa

**Author response:**

 We thank the reviewer for the recommendation to expand our discussion of biological and psychosocial mechanisms and alternative hypotheses related to the role of exercise in PTSD symptom modulation. However, on submission, we were required to limit the literature review to under 500 words and reduce the Discussion from 2,155 to 1,239 words. Given the word limit constraints, we prioritized key findings. We recognize the need to further engage with alternative mechanisms (e.g., distraction vs. potential exacerbation of intrusive memories).

 We have taken note of the reviewer’s request to expand the introduction and discussion. In order to meet journal requirements, we have added the additional text to the OSF folder, given that we cannot extend these sections in the manuscript itself (https://osf.io/cxyjw). This has been indicated in the manuscript, at the end of the introduction section: “ Please refer to the Extended data for the Expanded introduction and discussion section.” (pg 7).

 Regarding the exclusion of human analogue studies (e.g., trauma film, fear conditioning, extinction paradigms) and the limitation to Single Prolonged Stress (SPS) models in non-human studies, we explicitly pre-specified our selection criteria. Specifically, we limited eligibility to controlled human and non-human studies. Further, we restricted non-human studies to SPS models as it is the most widely used paradigms in non-human studies of PTSD. Moreover, studies have shown that the SPS model in animals and human PTSD involve experiencing traumatic events that can lead to similar behavioural and physiological changes. Since our last literature search, we have conducted five updates, with no new eligible studies identified. We have provided details for updating the systematic review and stopping the living mode of the review in the protocol (https://wellcomeopenresearch.org/articles/8-494/v2).

 “The literature search will be updated every three months, and any new studies meeting the inclusion criteria will be incorporated into the latest version of the evidence. If sufficient new data exists, a triangulation meeting will be held to incorporate the new evidence. Furthermore, it will be decided whether the review updates will continue, and whether to widen the scope of the review (this may include contacting authors of major studies to provide missing data). When there is insufficient new data to warrant triangulation, the review will be updated again after three months”. We appreciate the suggestion to make our included study list for human studies more accessible. We have now added a complete reference list of included studies in the Extended Data (https://osf.io/qdmxz): “ Please refer to the Extended data for the list of eligible human studies.” (pg 11) It is important to note in our inclusion criteria: “We excluded exercise interventions that incorporated mindfulness (e.g., Pilates, martial arts, yoga, tai chi, and horseback riding) or psychotherapy-based techniques into the exercise protocol.” (pg 8)  

# Reviewer report: Reviewer response for version 1

**DOI:** 10.21956/wellcomeopenres.25365.r116285

**Published:** 2025-01-10

**Authors:** Ryan S Garten

**Affiliations:** 1 Virginia Commonwealth University, Richmond, Virginia, USA

The submission was clear and well executed. The findings are interesting. More detail regarding the exact exercise parameters for the studies chosen (e.g. intensity, duration, time. etc.) and analysis of other PTSD-linked symptoms (anxiety and depression) in the human model would add strength to this manuscript. For the latter, individuals with PTSD may report similar triggers but have lower anxiety and depressive responses to them likely resulting in an improvement in health outcomes in the long term. Also, some exercise metric should be included to show that the exercise had it's desired effect. Specifically, did the exercise program increase exercise tolerance (maximal oxygen consumption, strength, endurance, etc.). This would indicate if exercise regimens were effective with the opposite (poorly prescribed exercise leading to no exercise-specific improvements) may explain why no improvements were reported in PTSD symptoms.

Are the rationale for, and objectives of, the Systematic Review clearly stated?

Yes

Is the statistical analysis and its interpretation appropriate?

Yes

If this is a Living Systematic Review, is the ‘living’ method appropriate and is the search schedule clearly defined and justified? (‘Living Systematic Review’ or a variation of this term should be included in the title.)

Yes

Are sufficient details of the methods and analysis provided to allow replication by others?

Yes

Are the conclusions drawn adequately supported by the results presented in the review?

Yes

Reviewer Expertise:

My expertise is in exercise physiology with a focus on areas such as cardiovascular physiology and redox balance.

I confirm that I have read this submission and believe that I have an appropriate level of expertise to confirm that it is of an acceptable scientific standard, however I have significant reservations, as outlined above.

# Response

**Published:** 2025-01-31

**Authors:** Simonne Wright87

**Affiliations:** Department of Psychiatry, Stellenbosch University, Stellenbosch, Western Cape, South Africa

**The submission was clear and well executed. The findings are interesting.** **More detail regarding the exact exercise parameters for the studies chosen (e.g. intensity, duration, time. etc.) **  

Author response  We have added to the results section “exercise intensity (moderate-intensity , 40-60% of Maximum Heart Rate, or high-intensity, >60% of Maximum Heart Rate)” (pg 9)   We have added in the results section: “ Follow-up outcome data were only reported in three studies (Bryant et al., 2023; Voorendonk et al., 2023; Young-McCaughan et al., 2022) and therefore insufficient for conducting a meta-analysis”. (pg 11)   We have provided the intervention detail for each study in our Extended data under “Study details for the included trials” ( https://osf.io/ezm7u).   In the methods section we have stated… “Data were collected at specific time points including at pre-intervention (baseline) and at the primary endpoint, defined as the first post-intervention time point or study endpoint if exercise was given for the entire duration of the trial. We also collected data for all subsequent follow-up assessment time points.” (pg 9)  

**and analysis of other PTSD-linked symptoms (anxiety and depression) in the human model would add strength to this manuscript. For the latter, individuals with PTSD may report similar triggers but have lower anxiety and depressive responses to them likely resulting in an improvement in health outcomes in the long term.**

Author response  Thank you for your valuable suggestion to include analyses of anxiety and depression in our study. We agree that exploring these PTSD-related symptoms will add depth and significantly enhance the manuscript. Based on your feedback, we have now incorporated anxiety and depression as secondary outcomes. The manuscript has been revised accordingly, with specific changes detailed below:   Under secondary outcomes we have added: “ … depression symptom severity, anxiety symptom severity…” (pg 9)   In the results section: “The Extended data provide a synthesis of the secondary outcomes (functional impairment, individual PTSD symptom clusters, loss of PTSD diagnosis , depression symptom severity, and anxiety symptom severity), for which there was insufficient data to conduct a meta-analysis.”  (pg 16)   The ‘Synthesis without a meta-analysis’ in the Extended data (https://osf.io/mhr8s) has been updated to include the depression symptom severity and anxiety symptom severity outcomes: “ **Depression symptom severity** Six studies examined the effects of exercise on depression symptom severity post-intervention (Greene & Petruzzello, 2022; Nordbrandt et al., 2020a; Rosenbaum et al., 2015a; Voorendonk et al., 2023; Whitworth et al., 2019a; Whitworth et al., 2019b). Voorendonk et al. (2023) observed large and significant reductions in depression symptom severity in both the exercise + therapy, and the attention control + therapy conditions. No significant differences between the two groups were found. Similarly, Bryant et al. reported a decrease in depression symptoms across both groups but found no significant advantage for exercise + therapy condition compared to the attention control + therapy condition (SMD = 0.2; 95% CI [−0.1, 0.7]). Nordbrandt et al. (2020) reported comparable pre-to-post-treatment improvement in both groups, suggesting no added benefit of exercise for reducing depression symptoms. In the two studies by Whitworth et al. (2019a, 2019b), neither reported a significant between-group difference in depression symptoms post-intervention. In contrast, Rosenbaum et al. (2015) found significantly greater improvement in depression symptoms in the exercise + TAU condition compared to TAU only condition (MD = −7.0; 95% CI [−11.9, −2.1], with a large effect size (partial eta-squared = 0.15). Green et al. (2022) also noted a reduction in depressive symptoms across all groups, including high-intensity functional exercise (d = 0.50), moderate-intensity circuit exercise (d = 1.03), and the control condition (d = 0.34), without clear evidence favouring the exercise interventions. Overall, the evidence suggests that while exercise interventions are associated with a significant reduction in depression symptom severity, these improvements are generally comparable to those observed in non-exercise-based treatments. No consistent superiority of exercise over other interventions in reducing depression symptoms was found, though some studies, such as Rosenbaum et al. (2015), reported notable benefits. **Anxiety symptom severity** Seven studies examined the effects of exercise on anxiety symptom severity post-intervention (Bryant et al., 2023; Greene & Petruzzello, 2022; Nordbrandt et al., 2020b; Rosenbaum et al., 2015b; Voorendonk et al., 2023; Whitworth et al., 2019a; Whitworth et al., 2019b) Voorendonk et al. (2023) reported a substantial reduction in anxiety symptom severity post-intervention in both the exercise + therapy, and the attention control + therapy conditions, with no significant differences between the two. Similarly, Bryant et al. (2023) observed a significant decrease in anxiety symptoms in both groups but found no significant advantage in the exercise + therapy condition compared to the attention control + therapy condition (SMD = 0.3; 95% CI [−0.1, 0.6]). Rosenbaum et al. (2015) showed that participants in  the exercise + TAU condition experienced a significantly greater improvement in anxiety symptoms compared to TAU only condition (MD = −6.3; 95% CI [−10.3, −2.3], p = 0.003), with a large effect size (partial eta-squared = 0.17). Similarly, Nordbrandt et al. (2020) reported a comparable pre-to-post-treatment reduction in anxiety symptoms in both groups, indicating no clear advantage for exercise. While Whitworth et al. (2019a) found no significant between-group difference in anxiety symptoms post-intervention, Whitworth et al. (2019b) identified a significant reduction in anxiety symptoms in participants in the exercise-only condition compared to the attention control condition. Green et al. (2022) further noted that state anxiety significantly decreased immediately following moderate-intensity circuit exercise (d = 0.57) and sedentary activity (d = 0.64), but not high-intensity exercise (d = 0.24). Overall, the findings suggest that while exercise interventions can effectively reduce anxiety symptom severity, their benefits are often comparable to those achieved with non-exercise-based treatments. Although some studies, such as Rosenbaum et al. (2015) and Whitworth et al. (2019b), highlight significant advantages of specific exercise types, the evidence does not consistently demonstrate the superiority of exercise interventions **.**”   We have noted this addition in the “Protocol deviations” document in the Extended data (https://osf.io/a4e6f) “ 3. Expanded Secondary Outcome Measures: Depression symptom severity and anxiety symptom severity were added as secondary outcomes.”  

**Also, some exercise metric should be included to show that the exercise had it's desired effect. Specifically, did the exercise program increase exercise tolerance (maximal oxygen consumption, strength, endurance, etc.). This would indicate if exercise regimens were effective with the opposite (poorly prescribed exercise leading to no exercise-specific improvements) may explain why no improvements were reported in PTSD symptoms.**

Author response  Unfortunately, not all studies included in our systematic review reported objective measures of exercise tolerance or fitness improvements. Moreover, the metrics provided varied considerably across studies, complicating meaningful comparisons. This inconsistency and lack of standardized data limit our ability to comprehensively evaluate the relationship between physiological adaptations to exercise and changes in PTSD symptoms.   To address this, we have added a new document to the Extended data, titled ‘Summary of Exercise Metrics Post-Intervention’ (https://osf.io/f27bh). This document consolidates and details the available information on exercise metrics reported in the studies.   Additionally, as noted in the **‘Results of the RoB for PTSD Severity Outcome in the Extended Data’ **(https://osf.io/hm6y8), only one study was rated as having a low risk of bias for **Domain 2** (bias due to deviations from intended interventions).   We have added to the results section: “ Findings from the included studies demonstrate variable adherence to exercise protocols, with some reporting improvements in metrics such as muscular strength and weekly energy expenditure, while others note minimal differences between the exercise and comparison groups. Additionally, several studies did not report between-group differences in exercise outcomes, further complicating the interpretation of the relationship between exercise and PTSD outcomes. Detailed information on post-intervention exercise metrics reported in the studies is presented in the Extended data” (pg 11)   In limitations…  “ A notable limitation of the studies included is the lack of consistent reporting on objective measures of exercise tolerance or fitness improvements between groups, This inconsistency not only limits the ability to assess the effectiveness of the prescribed exercise regimens but also obscures the potential relationship between physiological adaptations and PTSD symptom changes. To address this gap, future research should prioritize the inclusion of standardized, objective metrics of exercise when reporting outcomes. These measures could provide critical insights into whether exercise programs are being optimally implemented and whether improvements in physical fitness contribute to the therapeutic benefits for PTSD.” (pg 21)   Bryant, R. A., Dawson, K. S., Azevedo, S., Yadav, S., Cahill, C., Kenny, L., Maccallum, F., Tran, J., Rawson, N., Tockar, J., & et al. (2023). Augmenting trauma-focused psychotherapy for post-traumatic stress disorder with brief aerobic exercise in Australia: a randomised clinical trial [Journal article]. *The lancet. Psychiatry*, *10*(1), 21‐29. https://doi.org/10.1016/S2215-0366(22)00368-6 Voorendonk, E. M., Sanches, S. A., Tollenaar, M. S., Hoogendoorn, E. A., de Jongh, A., & van Minnen, A. (2023). Adding physical activity to intensive trauma-focused treatment for post-traumatic stress disorder: results of a randomized controlled trial. *Front Psychol*, *14*, 1215250. https://doi.org/10.3389/fpsyg.2023.1215250 Young-McCaughan, S., Peterson, A. L., Mintz, J., Hale, W. J., Dondanville, K. A., Borah, E. V., Blount, T. H., Blankenship, A. E., Fina, B. A., Hall-Clark, B. N., Hernandez, A. M., Jacoby, V. M., Malach, S. L., Williams, J. M., Compton, K. E., Bingham, M. O., Vriend, C. A., Inman, A. W., Brundige, A., . . . Yarvis, J. S. (2022). Testing the role of aerobic exercise in the treatment of posttraumatic stress disorder (PTSD) symptoms in U.S. active duty military personnel: a pilot study [Article]. *Cognitive Behaviour Therapy*, *51*(4), 309-325. https://doi.org/10.1080/16506073.2021.2001689
